# Demographics of Fall-Related trauma among the Elderly Presenting to Emergency Department; a Cross-Sectional Study

**DOI:** 10.22037/emergency.v5i1.18497

**Published:** 2017-09-12

**Authors:** Hamid Reza Morteza Bagi, Sajjad Ahmadi, Maryam Hosseini

**Affiliations:** 1 Emergency Medicine Research Team, Tabriz University of Medical Sciences, Tabriz, Iran.; 2 Emergency Medicine Department, Maragheh University of Medical Sciences, Maragheh, Iran.; 3 Faculty of Medicine, Tabriz University of medical Sciences, Tabriz, Iran.

**Keywords:** Trauma severity indices, accidental falls, aged, demography, cross-sectional studies

## Abstract

**Introduction:**

Falling is reported to be the most common cause of mortality due to trauma in individuals over the age of 75 years. The present study is designed with the aim of determining the demographics of fall-related trauma among the elderly presenting to emergency department (ED).

**Methods:**

The present prospective cross-sectional study was carried out on all elderly patients ≥ 60 years old presenting to ED of a major referral trauma center in North West of Iran during 1 year. Demographic data, location and height of falling, duration of hospitalization, trauma severity and in-hospital outcome of the patients were gathered and reported via descriptive statistics.

**Results:**

228 patients with the mean age of 70.96 ± 5.2 years were studied (53.9% female). Most patients were in the 66-70 years age range (32.6%) and had a history of hypertension (22.3%), who had visited following a fall inside the house (69.3%), due to slipping (73.7%), and from a height equal to or less than 2m (71.9%). 6 (2.6%) patients died in the hospital. Mean trauma severity of patients based on ISS, RTS, and TRISS were 10.65 ± 3.95 (3-19), 7.84 ±.21 (1.4-14.5) and 1.66 ±1.31 (-1.49-3.82), respectively. Regarding need for hospitalization, only ISS shows a significant difference between outpatients and inpatients (p = 0.023). Patients who died had a significantly higher trauma severity based on ISS (p < 0.0001) and RTS (p < 0.0001).

**Conclusion:**

Based on the findings of the present study, slipping and syncope are the most common causes of falling in the studied elderly that had mostly happened inside the house and from a height less than 2m. Therefore, most patients were in the mild to moderate range of trauma severity. ISS and RTS were significantly higher in the 6 (2.6%) patients who died.

## Introduction

Increase in life expectancy has led to a rise in the number of elderly individuals all over the world and aging has become one of the most important challenges of public health in various countries. Due to their lower activity, old people are less likely to face injuries but if they get injured they have a higher mortality rate compared to young people because of disorders in physiological functions and body systems as well as presence of other medical problems. Currently, trauma is the 5^th^ cause of death among elderly people and falling is the most common cause of mortality due to trauma in individuals over the age of 75 years (1-3). In total, falling leads to about 40% of deaths due to trauma in old people (4).

20% to 28% of the elderly in Iran experience falling, and fear of falling leads to limited activity in 30% of old people (5, 6). Falling makes up 10-15% of emergency department (ED) visits (4, 7, 8) and its outcome in old people varies from complete recovery to death. Duration of hospitalization among the elderly also ranges from a few days to being permanently crippled (9-15).

It seems that planning on prevention of the accident is the best and most cost effective measure in this regard. However, any planning requires information on the epidemiologic characteristics and the situation of the subject of planning. Therefore, the present study is designed with the aim of determining the demographics of fall-related trauma among the elderly presenting to ED.

## Methods

***Study design and settings***

The present prospective cross-sectional study was carried out on elderly patients (≥ 60 years old) presenting to ED of Imam Reza hospital, Tabriz, Iran, following trauma caused by falling, from April 2015 to March 2016 (during 1 year). This hospital is one of the major referral trauma centers in North West of Iran. The study was approved by the ethics committee of Tabriz University of Medical Sciences and the researchers adhered to principles of Helsinki declaration and keeping patient data confidential. Oral consent was obtained from the patients for participating in the study.

**Table1:** Baseline characteristics of the studied patients

**Variable **	**Number (%)**
**Sex **	
Male	105 (46.1)
Female	123 (53.9)
**Age (year)**	
60 – 65	39 (17.1)
66 – 70	74 (32.6)
71 – 75	64 (28.1)
76 – 80	45 (19.7)
> 80	6 (2.6)
**Underlying disease**	
Hypertension	51 (22.3)
Ischemic heart disease	22 (9.6)
Osteoporosis	23 (10.1)
Diabetes	12 (5.2)
Neurologic disease	12 (5.2)
None	125 (54.8)
**Duration of hospitalization**	
Outpatient	88 (38.6)
< 5days	98 (43.0)
> 5 days	42 (18.4)

**Table 2:** Accident characteristics of the studied patients

**Variable **	**Number (%)**
**Location of fall**	
Outside the house	70 (30.7)
Bathroom	47 (20.6)
Kitchen	45 (19.7)
Steps	34 (14.9)
Other	32 (14.0)
**Cause of fall**	
Slipping	168 (73.7)
Altered level of consciousness	51 (22.4)
Other	9 (3.9)
**Height of fall (m)**	
≤ 2	134 (71.9)
> 2	49 (21.5)
Unknown	15 (6.6)

**Table 3:** Comparison of mean trauma severity between inpatients and outpatients as well as dead and alive patients based on ISS, RTS and TRISS indices

**Variable **	**Trauma severity (mean ** **±** ** standard deviation)**					
	**ISS**	**P value**	**RTS**	**P value**	**TRISS**	**P value**
**Hospitalization**						
Outpatient	9.9 ± 3.41	0.023	7.96 ± 2.42	0.295	1.52 ± 1.40	0.453
Inpatient	11.12 ± 4.20		7.64 ± 1.83		1.65 ± 1.23	
**Mortality **						
Alive	10.55 ± 3.92	0.0001	7.86 ± 2.23	0.0001	1.60 ± 1.31	0.406
Dead	20.66 ± 6.68		13.50 ± 1.00		2.05 ± 0.91	

*Participants*

All patients, 60 years old or older, who had visited the trauma unit of the mentioned ED during the study period following falling and were willing to participate were included via consecutive sampling. Patients who had died before arriving at the ED or were discharged against medical advice (71 patients), were excluded from the study.

*Data gathering*

A checklist consisting of baseline characteristics (age, sex, underlying illnesses), location of falling, height of falling, duration of hospitalization, data required for calculation of trauma severity based on Injury Severity Score (ISS), Revised Trauma Score (RTS), and Trauma and injury severity score (TRISS), and in-hospital mortality was filled for all the patients. The person in charge of data gathering and calculation of trauma severity was a senior emergency medicine resident, under supervision of the corresponding attend.

- ISS is calculated based on the severity of trauma to face, head, extremities, chest, and abdomen (13). If this score is equal to or higher than 16, the patient is deemed multiple trauma (14, 15). An ISS lower than 7, indicates mild physical injury, 7-12 moderate injury and more than 12 shows severe injury.
- In RTS, trauma severity is calculated based on level of consciousness, systolic blood pressure and respiratory rate and is also used for triage of the patients (16).
- TRISS indicates the prognosis of trauma patients and acts based on the physiologic and anatomic status of the patient on admission to ED. This system of determining trauma severity is in fact a combination of ISS and RTS (17).

*Statistical analysis*

Data were analyzed by SPSS version 21. Quantitative data are reported as mean and standard deviation and qualitative ones are reported as frequency and percentage. To compare 2 groups, t-test and chi square tests were used and to calculate trauma severity based on the mentioned indices, medical calculator was used. Significance level in analyses was considered to be less than 0.05.

## Results

***Baseline characteristics***

228 patients with the mean age of 70.96 ± 5.2 years were studied (53.9% female). Tables 1 and 2 depict the baseline characteristics of the patients and fall characteristics. According to these results, most patients were in the 66-70 years age range (32.6%) and had a history of hypertension (22.3%), who had visited following a fall inside the house (69.3%), due to slipping (73.7%), and from a height equal to or less than 2m (71.9%). 6 (2.6%) patients died in the hospital and others were discharged from ED (38.6%) or from surgery department after a few days of hospitalization (61.4%).

*Trauma severity*

Mean trauma severity of patients based on ISS, RTS, and TRISS were 10.65 ± 3.95 (3-19), 7.84 ±.21 (1.4-14.5) and 1.66 ±1.31 (-1.49-3.82), respectively. Trauma severity based on ISS was less than 7 in 60 (26.3%) patients, between 7 and 12 in 98 (43.0%) and over 12 in 70 (30.7%) injured patients. Table 3 compares mean trauma severity of the patients based on the 3 mentioned indices between patients who survived and those who died as well as outpatients and inpatients. As can be seen, only ISS shows a significant difference between outpatients and inpatients (p = 0.023). In addition, patients who died had a significantly higher trauma severity based on ISS (p < 0.0001) and RTS (p < 0.0001).

## Discussion

Based on the findings of the present study, slipping and syncope were the most common causes falling in the studied elderly, who had mostly fallen from a height less than 2m and inside the house. Most patients were in the mild to moderate range of trauma severity. ISS and RTS were significantly higher in the 6 (2.6%) patients who had died.

Statistics show that each year on average 28 to 35% of the elderly over 60 years old fall and in those over the age of 70 years this rate rises to 32 to 42% and in half of the cases, it happens recurrently. This emphasizes the necessity of taking preventive measures and applying plans to prevent this from happening again (18, 19).

In a study carried out in 2004 by Schoenfelder et al., falling rate was higher in women compared to men (82.10% versus 17.90%) (4). Swanenburg et al. in 2010 also reported the higher prevalence of this problem in women (83% vs. 17%) (20). However, in the present study affected men and women were relatively equal.

In Corsinovi et al. study on 620 elderly patients that were treated following a fall, no correlation was found between age and falling rate (14).

Regarding quantitative trauma severity, findings showed that mean ISS was 10.67 and most patients had moderate injury. In a study by Zare et al. evaluating trauma in emergency patients whose mean age was 29.60 years, the results showed that quantitative trauma severity was 16.98, which can show the higher severity of trauma and risky behaviors in young people compared to the elderly (21).

In 2009, Hariharan et al. evaluated the clinical value of TRISS in 326 trauma patients. Mean survival rate in adults was reported to be 98.50% in the study, which indicates 1.5% mortality in these patients. These researchers concluded that TRISS was not so accurate in predicting the prognosis of trauma patients (22). In addition in 2004, Murlidhar et al. evaluated TRISS in a study of trauma patients’ outcome and reported that the predicted mortality rate in patients by this index was 10.89%. This rate was 61.60% and 16.60% for RTS and ISS, respectively but the real mortality rate was 21.26%. The researchers reported the cause of this vast difference to be the higher age of the patients studied compared to other studies (23). Meanwhile, in another study conducted by Mitchell et al. in 2007 in Canada the ability of indices such as TRISS to predict the prognosis of trauma patients were evaluated and shown to be acceptable (24). 

The obtained results regarding trauma severity indices showed that TRISS did not correlate with hospitalization status and mortality of the patients; however, ISS correlated with hospitalization status and both ISS and RTS correlated with mortality of the patients.

Studies show that falling happens following a complex interference of risk factors that affect the health status of an elderly individual, directly or indirectly. These factors are categorized in 4 groups: biological, behavioral, environmental, and socioeconomic factors (18). Although in this study, these factors were not evaluated individually, the evidence shows that evaluating and controlling these factors is of great importance. 

Limitations

Small sample size, being single-centered and the evaluated variables being few could be counted as the limitations of this study. However, considering the limited number of these studies in Iran, the findings of this study can help in understanding the conditions ruling trauma due to falling in the elderly. 

## Conclusions

Based on the findings of the present study, slipping and syncope are the most common causes of falling in the studied elderly that had mostly happened inside the house and from a height less than 2m. Therefore, most patients were in the mild to moderate range of trauma severity. ISS and RTS were significantly higher in the 6 (2.6%) patients who died.
